# Bioconversion of lignocellulose: inhibitors and detoxification

**DOI:** 10.1186/1754-6834-6-16

**Published:** 2013-01-28

**Authors:** Leif J Jönsson, Björn Alriksson, Nils-Olof Nilvebrant

**Affiliations:** 1 Department of Chemistry, Umeå University, Umeå SE-901 87, Sweden; 2 Processum Biorefinery Initiative AB, Örnsköldsvik, SE-891 22, Sweden; 3 Borregaard, Sarpsborg, 1701, Norway

## Abstract

Bioconversion of lignocellulose by microbial fermentation is typically preceded by an acidic thermochemical pretreatment step designed to facilitate enzymatic hydrolysis of cellulose. Substances formed during the pretreatment of the lignocellulosic feedstock inhibit enzymatic hydrolysis as well as microbial fermentation steps. This review focuses on inhibitors from lignocellulosic feedstocks and how conditioning of slurries and hydrolysates can be used to alleviate inhibition problems. Novel developments in the area include chemical *in-situ* detoxification by using reducing agents, and methods that improve the performance of both enzymatic and microbial biocatalysts.

## Review

### Background

Lignocellulose provides an abundant renewable resource for production of biofuels, chemicals, and polymers [1-3]. Biorefineries, in which lignocellulosic biomass is converted to various commodities, are likely to become increasingly important in future society as complement and alternative to the oil refineries of today. Commodities produced from renewable resources offer an alternative to products based on dwindling supplies of petroleum and permit a move towards improved energy security and decreased impact on the environment. Lignocellulosic feedstocks include residues from agriculture and forestry, energy crops, and residues from biorefineries and pulp mills. Lignocellulosic biomass can contribute significantly to the future global energy supply without competition with increasing food demand for existing arable land [4].

Liquid biofuels include bioalcohols, such as ethanol and butanol, and biodiesel. Ethanol is the most important liquid biofuel of today. Bioalcohols are manufactured in fermentation processes, in which microbial biocatalysts, yeasts or bacteria, convert sugars to alcohols. The ethanol that is used today is mainly manufactured from sugar or starch-based raw materials. However, very large-scale use of bioalcohols in the energy sector will require production from lignocellulosic feedstocks [1-5], which have the added benefit that they are not used for food. This review focuses on biocatalyst inhibitors formed during acidic thermochemical pretreatment of lignocellulosic feedstocks, and how conditioning of slurries and hydrolysates can be used to alleviate inhibition problems connected with hydrolytic enzymes and the yeast *Saccharomyces cerevisiae*.

### Lignocellulose and pretreatment of lignocellulosic feedstocks

Lignocellulosic feedstocks mainly consist of cellulose, hemicellulose, and lignin [6,7]. Cellulose is an unbranched homopolysaccharide consisting of D-glucopyranosyl units. Hemicelluloses are branched heteropolysaccharides consisting of both hexose and pentose sugar residues, which may also carry acetyl groups. The third main component, lignin, consists of phenylpropane units linked together by different types of interunit linkages of which ether bonds are the most common. Lignocellulose polysaccharides are hydrolyzed to provide the monosaccharides used by microbial biocatalysts in fermentation processes. The crystalline parts of the cellulose are more resistant to hydrolysis than are the amorphous parts. Compared to starch, the polysaccharides of lignocellulose are more resistant to hydrolysis. Furthermore, woody biomass is generally more resistant to degradation than other types of lignocellulose. Softwood is typically more difficult to hydrolyze than hardwood or agricultural residues [8-12].

**Figure 1:**
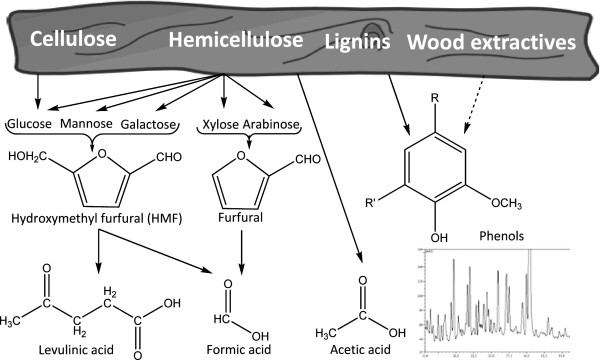
**Formation of inhibitors. **Scheme indicating main routes of formation of inhibitors. Furan aldehydes and aliphatic acids are carbohydrate degradation products, while lignin is the main source of phenolic compounds, as indicated by guaiacyl (4-hydroxy-3-methoxyphenyl) and syringyl (4-hydroxy-3,5-dimethoxyphenyl) moieties found in many phenolics. While the contents of furan aldehydes and aliphatic acids are relatively easy to determine, the quantification and identification of phenolic compounds remain challenging. The insert shows the variety of peaks representing phenolic compounds found in a hydrolysate of Norwegian spruce, as indicated by analysis using liquid chromatography-mass spectrometry (LC-MS).

Hydrolysis of cellulose can be catalyzed by using strong inorganic acids or hydrolytic enzymes, including cellulases [13,14]. Acid hydrolysis of cellulose requires severe conditions. Enzymatic hydrolysis is often considered as the most promising approach for the future [5]. Lignocellulosic biomass intended for production of liquid biofuels is typically pretreated in an acidic thermochemical process step to increase the susceptibility of the cellulose to enzymatic hydrolysis [5,9,12]. The pretreatment usually degrades the hemicellulose leading to the formation of products such as pentose and hexose sugars, sugar acids, aliphatic acids (primarily acetic acid, formic acid and levulinic acid), and furan aldehydes [5-hydroxymethylfurfural (HMF) and furfural] (Figure 1). After hydrolysis of lignocellulose polysaccharides, lignin remains as a solid residue, although a minor part is degraded to phenolics and other aromatic compounds (Figure 1). Sugars derived from hemicelluloses will account for a substantial part of the total sugar and it is desirable that they are included in the subsequent fermentation step. The monosaccharides obtained through the hydrolysis process are then fermented by microbial catalysts to the desired product, most commonly ethanol produced with the yeast *S. cerevisiae*.

Hydrolysis and fermentation can be performed separately (separate hydrolysis and fermentation; SHF) or simultaneously (simultaneous saccharification and fermentation; SSF). Consolidated bioprocessing (CBP) refers to a process in which the fermenting microorganism also contributes by producing cellulolytic enzymes [15].

### Inhibitors of enzymatic and microbial biocatalysts

The generation of by-products from the pretreatment is strongly dependent on the feedstock and the pretreatment method. Substances that may act as inhibitors of microorganisms include phenolic compounds and other aromatics, aliphatic acids, furan aldehydes, inorganic ions, and bioalcohols or other fermentation products. Examples of inhibitory fermentation products are ethanol and butanol. As most microorganisms, *S. cerevisiae* is inhibited by butanol concentrations in the range 1-2% (v/v) [16], but it is able to withstand much higher concentrations of ethanol. In high-gravity alcoholic fermentations, *S. cerevisiae* produces ethanol concentrations of 17% (v/v) or higher [17]. Hydrolytic enzymes are inhibited by their products, i.e. sugars such as cellobiose and glucose [18], by fermentation products such as ethanol [19,20], and by phenolic compounds [21].

### Aromatic compounds

A large number of different phenolic compounds are formed from lignin during acid-catalyzed hydrolysis or pretreatment of lignocellulose. Phenolic compounds and other aromatics are formed during pretreatment regardless of whether an acid catalyst is added to the reaction [22]. Carboxylic acids formed during the pretreatment will contribute to the formation of an acidic environment. Furthermore, some extractives are phenolic compounds [6,7]. Formation of phenolic compounds from sugars is another possibility [23], although the significance of this route remains to be investigated.

Different analytical techniques, primarily gas chromatography–mass spectrometry (GC-MS) and liquid chromatography-mass spectrometry (LC-MS), have been used to identify specific aromatic compounds in acidic hydrolysates from various kinds of lignocellulosic feedstocks, such as corn stover [24-26], oak [27], pine [26,28,29], poplar [24,30-32], spruce [33-35], sugarcane bagasse [22], switchgrass [24], and willow [36]. In addition, aromatic degradation products in hydrolysates produced by alkaline methods have been investigated [26,37]. The large number and the diversity of the aromatic compounds found in different lignocellulose hydrolysates (Figure 1) make identification and quantification of separate compounds complicated. Group analysis of phenolic compounds offers an alternative approach. GC-MS has been used to estimate the total amount of phenols in lignocellulose hydrolysates [33,36]. The total amount of phenols in a spruce wood hydrolysate was determined spectrophotometrically by using the Prussian Blue method [33]. Persson et al. [34] compared the Prussian Blue method with another spectrophotometric method, based on Folin-Ciocalteu's reagent, and found that the latter gave more reliable results with respect to analysis of phenolic compounds in the hydrolysate. A peroxidase-based biosensor was also tested, as an alternative to the spectrophotometric methods [34]. Furthermore, a method for group analysis of phenols by high-performance liquid chromatography (HPLC) has also been used [38]. Although the Folin-Ciocalteu method is the most convenient approach to analyze the total phenolic contents in lignocellulose hydrolysates, it should be avoided in experiments with redox reagents (such as reduced sulfur compunds including dithionite, dithiothreitol, and sulfite), in which the HPLC method serves as a better option [39]. It should also be noticed that phenol analysis using the Folin-Ciocalteu reagent is related to the Lowry method for determination of the total protein content [40] and that it is therefore sensitive to potential media components such as hydrolytic enzymes, cell extracts, and hydrolyzed protein.

The effects of phenolics and other aromatic compounds, which may inhibit both microbial growth and product yield, are very variable, and can be related to specific functional groups [30,41]. In many cases, the mechanism of toxicity has not been elucidated. One possible mechanism is that phenolics interfere with the cell membrane by influencing its function and changing its protein-to-lipid ratio [42]. *S. cerevisiae* can convert some inhibitory phenolics to less toxic compounds. For instance, coniferyl aldehyde is reduced to coniferyl alcohol and dihydroconiferyl alcohol [41].

The role of phenolic inhibitors has been investigated using enzymic catalysts that specifically affect phenolic compounds without changing the concentrations of other inhibitors, such as aliphatic acids and furan aldehydes [33,36,43-45]. Enzymes, such as laccases and peroxidases, oxidize phenols to radicals that undergo coupling to larger molecules that are less toxic to fermenting microbes such as yeast [36].

Phenolic compounds are also investigated with regard to inhibition of enzymatic hydrolysis of cellulose [21]. Experiments with phenols suggest that one way in which they affect proteins is by inducing precipitation [46].

### Aliphatic acids

Lignocellulose hydrolysates contain aliphatic acids, such as acetic acid, formic acid, and levulinic acid. Acetic acid is formed primarily by hydrolysis of acetyl groups of hemicellulose, while formic acid and levulinic acid arise as acid-catalyzed thermochemical degradation products from polysaccharides (Figure 1). Formic acid is a degradation product of furfural and HMF (5-hydroxymethylfurfural), while levulinic acid is formed by degradation of HMF [47]. The pKa value of formic acid (3.75) is considerably lower than those of acetic acid (4.76) and levulinic acid (4.64). The toxic effect on *S. cerevisiae* is attributed to the undissociated form and increases in the order acetic acid < levulinic acid < formic acid. Inhibition of yeast was found to be apparent at concentrations exceeding 100 mM [48]. However, lower concentrations than 100 mM gave higher ethanol yields than fermentations with no aliphatic acids included [48]. The contents of aliphatic acids in slurries and hydrolysates vary strongly depending on the feedstock and the severity of the pretreatment. Feedstocks with high content of acetylated xylan, typically agricultural residues and hardwood, give higher concentrations of aliphatic acids than softwood. The total content of aliphatic acids in softwood hydrolysates is often below 100 mM and consequently beneficial for the ethanol yield rather than harmful [48,49].

Undissociated acids enter the cell through diffusion over the cell membrane and then dissociate due to the neutral cytosolic pH [50]. The dissociation of the acid leads to a decrease in the intracellular pH, which may lead to cell death. Alternatively, it may lead to increased ethanol yield at the expense of biomass formation as a consequence of the cell's attempt to maintain a constant intracellular pH by pumping out protons through the plasma membrane ATPase [51-53].

A group of compounds that can be mentioned in this context are uncouplers, i.e. amphiphilic molecules that dissolve in the inner mitochondrial membrane of eukaryotic cells and that have the ability to transfer protons across the membrane. By disrupting the proton gradient over the inner mitochondrial membrane, they disconnect the linkage between the respiratory chain and the oxidative phosphorylation that regenerates ATP from ADP. This mechanism differs from that proposed for aliphatic acids like acetic acid, as it inhibits the regeneration of ATP in mitochondria rather than stimulate the consumption of ATP at the plasma membrane. Some aromatic carboxylic acids may act as uncouplers, as has been shown in experiments with plant cells and salicylic acid [54], a compound that is also found in lignocellulose hydrolysates [26,36]. Another aromatic carboxylic acid, *p*-hydroxybenzoic acid, which is common in lignocellulose hydrolysates, did not exhibit the uncoupling effect observed for salicylic acid [54].

### Furan aldehydes

The furan aldehydes furfural and HMF, which also are commonly found in lignocellulose hydrolysates, are formed by dehydration of pentose and hexose sugars, respectively (Figure 1). Furfural and HMF inhibit the growth of yeast and decrease ethanol yield and productivity [48,55,56]. Under anaerobic conditions, *S. cerevisiae* can convert furfural to furfuryl alcohol [57,58] and HMF to 2,5-bis-hydroxymethylfuran [59]. Reduction of furfural has been linked to the co-factor NADH, while reduction of HMF has been found to be associated with consumption of NADPH [60]. A moderate addition of furfural to the growth medium was found to lead to increased ethanol yields for recombinant xylose-utilizing *S. cerevisiae* transformants [60]. This can be explained by the reduction of furfural to furfuryl alcohol, which will lead to a decreased formation of the undesirable by-product xylitol and an increased formation of ethanol. Model fermentations with furan aldehydes added to the medium suggest that yeast can tolerate quite high concentrations of furan aldehydes [48,61]. Martinez et al. [62] noticed that it took an addition of three times the original concentrations of the furan aldehydes to restore the inhibition of *E. coli* by a detoxified bagasse hydrolysate. These observations suggest that the inhibition might be due to other inhibitors present in the hydrolysate, other yet unidentified compounds, or perhaps to synergistic effects involving furan aldehydes. The capability of the microorganism to reduce furan aldehydes to the less toxic corresponding alcohols during fermentation in a bioreactor is sometimes referred to as *in-situ* detoxification [63]. The concept of biological *in-situ* detoxification is based on the presumption that it is the mere presence of the inhibitory substance that is the problem, rather than its bioconversion.

### Inorganic compounds

Inorganic ions that are present in lignocellulose hydrolysates originate from the lignocellulosic feedstocks, from chemicals added during pretreatment, conditioning and hydrolysis, and possibly from process equipment. The addition of salts results in a higher osmotic pressure, which may result in inhibitory effects [64,65]. At moderate concentrations, there is a possibility that inorganic ions enhance ethanol production in a similar way as moderate concentrations of aliphatic acids do. The proposed mechanism is increased demand of ATP due to increased transport over the plasma membrane. Extra ATP is acquired by an increased ethanol production at the expense of biomass formation.

*S. cerevisiae* is relatively salt tolerant compared to other yeasts, such as *Schizosaccharomyces pombe* and *Scheffersomyces* (*Pichia*) *stipitis*, but less tolerant than several *Candida* species [64]. In glucose-based medium, *S. cerevisiae* is capable to grow in a 1.5 M solution of sodium chloride. However, a more important factor than the absolute concentration of sodium is the intracellular ratio of Na^+^/K^+^, which preferably should be kept low. Maiorella et al. [66] investigated the effects of different salts on *S. cerevisiae* and found that the inhibition decreased in the following order: CaCl_2_, (NH_4_)_2_SO_4_ > NaCl, NH_4_Cl > KH_2_PO_4_ > MgCl_2_ > MgSO_4_ > KCl.

### Other inhibitory effects

Ethanol generated during fermentation inhibits viability, growth, glucose transport systems, and proton fluxes of *S. cerevisiae*. The yeast plasma membrane is affected with respect to permeability, organization, and lipid composition [67]. However, the ethanologenic microbes *S. cerevisiae* and *Zymomonas mobilis* can tolerate ethanol concentrations up to 18 and 12%, respectively [68]. The engineering of microbes for improved resistance to bioalcohols and other biofuels has recently been reviewed [16].

Potential synergistic effects of inhibitors have been studied in experiments with yeast and bacteria [69-71]. The results of these studies indicate synergistic effects of combinations of acids and furan aldehydes, as well as of combinations of different phenolics.

### Strategies to counteract inhibition problems

Several alternative measures can be taken to avoid problems caused by inhibitors. The concentrations of inhibitors and sugars in hydrolysates depend on the feedstock as well as on the conditions during pretreatment and hydrolysis [9,48]. Therefore, one possibility is to select less recalcitrant feedstocks and to utilize mild pretreatment conditions. However, it is desirable to utilize different varieties of lignocellulose if production of commodities from renewables should make a major impact on the market for fuels, chemicals, and materials. Furthermore, production of bulk chemicals is yield dependent, which implies that it is not reasonable to accept a poor sugar yield, and consequently a poor overall product yield, due to the use of insufficient pretreatment conditions.

It is also possible to design the fermentation process to avoid problems with inhibition, for example by using SSF to avoid inhibition of cellulolytic enzymes by sugars, or by using fed-batch or continuous cultivation rather than batch processes [72]. High yield and productivity, high product titer, and possibilities to recirculate process water are, however, important aspects of the chosen design. Ethanol production from diluted hydrolysates with low sugar content is associated with a high operating cost due to a more expensive distillation process [68].

There is a variety of different chemical, biological and physical methods that can be used to detoxify slurries and hydrolysates [33,73,74]. Approaches that have been studied include overliming and treatments with other chemicals, liquid-liquid extraction, liquid–solid extraction, heating and evaporation, and treatments with microbial and enzymatic biocatalysts (Table 1). Comparisons of different methods for detoxification, or conditioning, indicate that they differ significantly with respect to effects on hydrolysate chemistry and fermentability [33,75]. A common objection against detoxification is based on the assumption that it would require a separate process step.

**Table 1:** Techniques for detoxification of lignocellulose hydrolysates and slurries

**Technique**	**Procedure**	**Example**^a^
Chemical additives	Alkali [such as Ca(OH)_2_, NaOH, NH_4_OH]	[76,77]
	Reducing agents [such as dithionite, dithiothreitol, sulfite]	[39]
Enzymatic treatment	Laccase	[36,45]
	Peroxidase	[36]
Heating and vaporization	Evaporation	[33]
	Heat treatment	[78]
Liquid-liquid extraction	Ethyl acetate	[24,75]
	Supercritical fluid extraction [such as supercritical CO_2_]	[34]
	Trialkylamine	[79]
Liquid–solid extraction	Activated carbon	[80]
	Ion exchange	[38,81]
	Lignin	[82]
Microbial treatment	*Coniochaeta ligniaria*	[83,84]
	*Trichoderma reesei*	[33,85]
	*Ureibacillus thermosphaericus*	[86]

^a^The table includes one or two examples of each procedure (references are not exhaustive). Dilution, washing of solid fractions, and techniques based on the fermenting microbe are not included.

There are a number of strategies that concern the fermenting microorganism. The use of large inocula decreases inhibition problems [55,73,75]. However, the use of large inocula is considered to be a less attractive solution in an industrial context [87]. Using a large inoculum would be a possibility if the microorganism can be recirculated and reused at a reasonable cost. However, if the used fermentation broth contains a lot of solids, the separation of the microorganism could become a tedious task. This is the case in SSF processes, and as a consequence the use of fresh inocula is considered instead of recycling the microorganism [88].

Other possibilities that target the microorganism include selection of microbial species and strains that exhibit resistance to inhibitors. Adaptation of the microorganism to an inhibiting environment, possibly after inducing variation by mutagenesis, serves as an alternative option. Furthermore, genetic engineering can be employed to obtain transformed hyperresistant microbes. *S. cerevisiae* has been engineered for increased resistance to fermentation inhibitors by overexpression of enzymes conferring improved resistance to phenolics [89,90], furan aldehydes [91,92], and aliphatic acids [93,94]. Furthermore, overexpression of a transcription factor, Yap1 [95], and of multidrug-resistance proteins [95] has also generated hyperresistant *S. cerevisiae* transformants. In some of these cases, hyperresistance to lignocellulose hydrolysates has also been demonstrated [89,90,95].

Most of the studies on inhibition have had focus on the fermenting microorganism, while strategies that decrease inhibition of enzymes so far have received relatively little attention. Since most enzymatic hydrolysis processes involve mixtures of a pretreatment liquid and a solid cellulosic material, there are good reasons to take enzyme inhibition into account. Chemical detoxification, a powerful strategy to deal with inhibitor problems which also addresses enzyme inhibition, will be considered in more detail below.

### Chemical treatment

**Figure 2:**
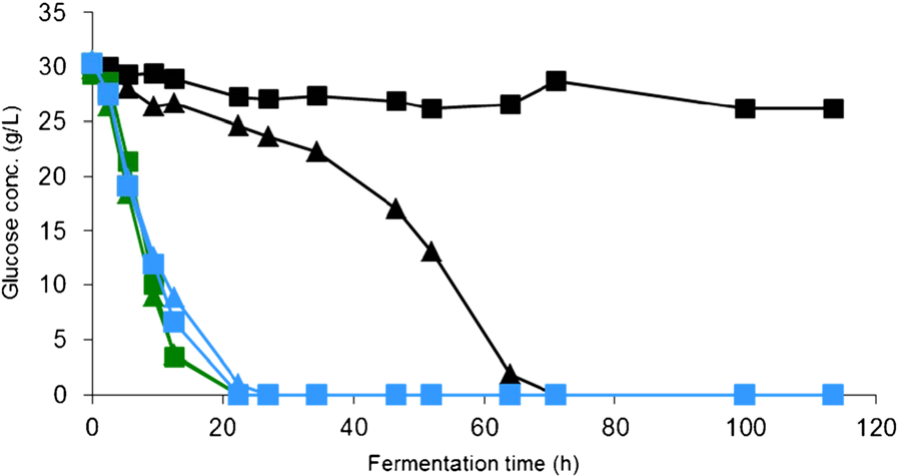
**Effects of genetic engineering for hyperresistance and chemical detoxification through alkaline treatment. **Ethanol production by *S. cerevisiae* (control transformant and transformant overexpressing Yap1 [95]): in spruce hydrolysate medium (black triangle, Yap1 transformant; black square, Control transformant), in alkali-detoxified spruce hydrolysate (green triangle, Yap1 transformant; green square, Control transformant), and in inhibitor-free medium (blue triangle, Yap1 transformant; blue square, Control transformant).

Although methods such as liquid-liquid extraction, ion exchange, and treatment with biocatalysts remain frequently studied options for detoxifying hydrolysates or slurries, the focus of this section will be detoxification by addition of alkali or other chemical agents. In comparisons of detoxification methods, treatment with calcium hydroxide (overliming) has emerged as one of the most efficient methods [33,75]. In many cases, overliming also seems to be the most economical choice [78]. Although biotechnical methods (reviewed in [74,96]) are very promising in a longer perspective, they are seldom compared to conventional methods, such as alkaline detoxification. A comparison between the performance of a hyperresistant *S. cerevisiae* transformant overexpressing Yap1 [95] and the effect of alkaline detoxification is shown in Figure 2. The result indicates that both approaches have a very clear positive impact, but only the fermentation after alkaline detoxification reaches a similar level as that of the reference fermentation.

Overliming of hydrolysates produced by pretreatment of lignocellulose with sulfuric acid results in the precipitation of calcium sulfate (gypsum) [76,97]. This keeps the concentration of soluble salts at a low level, which is favorable for the fermentation process [76,97]. However, treatment of hydrolysates with other types of alkali, such as ammonium hydroxide, can result in a fermentability that is equal to or even better than that of hydrolysates treated with overliming [76].

Although the mechanism of overliming is still not completely elucidated, considerable progress has been made. Van Zyl et al. [98] suggested that the detoxification effect of overliming was due to precipitation of toxic substances. Persson et al. [35] collected and analyzed precipitated material as well as the chemical composition of alkali-treated hydrolysates and concluded that the detoxification effect was due to chemical conversion rather than to removal of precipitated inhibitors. Furthermore, a comparison of different types of alkali for treatment of hydrolysates showed that it was possible to obtain an excellent ethanol yield (better than in a reference fermentation with similar sugar content but without inhibitors) after treatment with sodium hydroxide [77]. Since the treatment with sodium hydroxide did not give rise to any precipitate, this finding confirmed the conclusions drawn regarding the effects of alkaline treatment [35].

**Figure 3:**
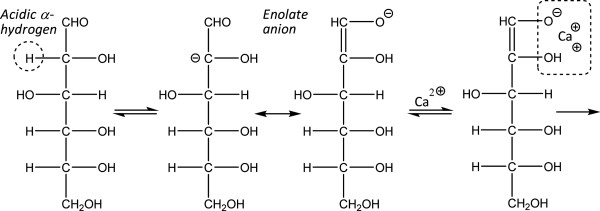
**Monosaccharide degradation in alkali. **Initial phase of degradation of glucose during alkaline treatment. Calcium ions stabilize the reactive enol intermediate, which in turn is degraded to HMF, and further to formic and levulinic acids.

A problem associated with alkali detoxification is that not only inhibitors are affected by the treatment, but also the sugars, which could lead to reduced ethanol yields (Table 2). Nilvebrant et al. [99] studied the effects of treatment time, temperature, and pH during alkali treatment of a spruce hydrolysate. During treatment with alkali, xylose was slightly more easily degraded than the other monosaccharides. Using similar conditions (time period, pH, and temperature), the effect of calcium hydroxide was larger than that of sodium hydroxide. More extensive sugar degradation during alkaline treatment by overliming can be attributed to the stabilisation of reactive enolate intermediates by calcium ions (Figure 3). The examples in Table 2 indicate that too harsh conditions result in extensive sugar degradation, which also has an adverse effect on ethanol production. However, it is also evident that a considerable improvement of the fermentability can be gained with a very small loss of sugar (about 1%) (Table 2) indicating that sugar loss is not always a valid objection to alkaline detoxification.

**Table 2:** Effects of alkaline treatment on monosaccharides and ethanol production

**System studied**	**Detoxification conditions**	**Improvement in fermentability**	**Effect on inhibitors and sugar**	**Reference**
Spruce hydrolysate. *S. cerevisiae*	Ca(OH)_2 _pH 10, 1 h	BEY^a^= 98%	Furan aldehydes, decrease: ~21%	[33]
		BEY^a ^(untreated^b^)= 71%	Phenols, decrease: ~19%	
		BEY^a ^(reference^c^)= 100%	Sugar, decrease: ~4%^d^	
	NaOH pH 10, 1 h	BEY^a^= 94%	Furan aldehydes, decrease: ~18%	
		BEY^a ^(untreated^b^)= 71%	Phenols, decrease: ~18%	
		BEY^a ^(reference^c^)= 100%	Sugar, decrease: ~4%^d^	
Bagasse hydrolysate. *E. coli*	Ca(OH)_2 _pH 9, 60°C, 0.5 h	Q _(24 h)_^e^: ~1.3 g/Lh	Furan aldehydes, decrease: ~69%	[97]
		No reference fermentation	Phenols, decrease: ~35%	
			Sugar, decrease: ~15%^f^	
	Ca(OH)_2 _pH 10, 60°C, 0.5 h	Q_(24 h)_^e^: ~ 1.0 g/Lh	Sugar, decrease: ~33%^f^	
		No reference fermentation		
Spruce hydrolysate. *S. cerevisiae*	Ca(OH)_2 _pH 12, 60°C, 170 h	Q_(24 h)_^e^: ~ 0.3 g/Lh	Furan aldehydes, decrease: ~100%	[100]
		No reference fermentation	Phenols, increase: ~150%	
				Sugar, decrease: ~68%^g^	
	Ca(OH)_2 _pH 11, 25°C, 20 h	Q_(24 h)_^e^: ~ 0 g/Lh	Furan aldehydes, decrease: ~77%		
		Q_(48 h)_^e^: ~ 0.3 g/Lh	Phenols, decrease: ~8%		
		No reference fermentation	Sugar, decrease: <5%^g^		
Bagasse hydrolysate. *S. cerevisiae*	Ca(OH)_2 _pH 10, 1 h	BEY^a^= 92%	Furan aldehydes, decrease: >25%	[43]	
		BEY^a ^(untreated^b^)= 68%	Phenols, decrease: ~17%		
		BEY^a ^(reference^c^)= 100%	Sugar, decrease: ~1%^f^		
Spruce hydrolysate. *S. cerevisiae*	Ca(OH)_2 _pH 11, 30°C, 3 h	BEY^a^= 120%	Furan aldehydes, decrease: ~59%	[49]	
		BEY^a^ (untreated^b^)= 5%	Phenols, decrease: ~22%		
		BEY^a^ (reference^c^)= 100%	Sugar, decrease: ~14%^h^		
Spruce hydrolysate. *S. cerevisiae*	NH_4_OH pH 10, 22°C, 3 h	BEY^a^ =110%	Not determined	[76]	
		BEY^a ^(untreated^b^)= 10%			
		BEY^a ^(reference^c^)= 100%			
Spruce hydrolysate. *S. cerevisiae*	NaOH pH 9, 55°C, 3 h	BEY^a^ = 111%	Furan aldehydes, decrease: ~33%	[77]	
		BEY^a^ (untreated^b^) = 6%	Phenols, decrease: ~12%		
		BEY^a^ (reference^c^) = 100%	Sugar, decrease: ~9%^d^		
	NH_4_OH pH 9, 55°C, 3 h	BEY^a^ =120%	Furan aldehydes, decrease: ~33%		
		BEY^a^ (untreated^b^) = 7%	Phenols, decrease: ~13%		
		BEY^a^ (reference^c^) = 100%	Sugar, decrease: ~7%^d^		
Corn stover hydrolysate. *Z. mobilis*	Ca(OH)_2_ pH 9, 50°C, 0.5 h	No reference fermentation. OEY^i^ = 62%	Sugar, decrease: ~7%^j^	[101]	
	Ca(OH)_2_ pH 10, 50°C, 0.5 h	No reference fermentation. OEY^i^ = 70%.	Sugar, decrease: ~13%^j^		
	Ca(OH)_2_ pH 11, 50°C, 0.5 h	No reference fermentation. OEY^i^ = 59%	Sugar, decrease: ~29%^j^		

^a ^Balanced ethanol yield given in percent of a reference fermentation of a sugar solution. ^b ^Untreated hydrolysate. ^c^ Reference sugar solution. ^d ^Glucose, xylose, arabinose, galactose mannose, and cellobiose. ^e ^Ethanol productivity. ^f^ Glucose, xylose, arabinose, galactose, and mannose. ^g^ Glucose, xylose, galactose, and mannose. ^h ^Glucose and mannose. ^i ^Overall ethanol yield, yield calculated on sugars present prior to detoxification, given in percent of the theoretical yield. ^j^ Glucose, xylose, and arabinose.

Ethanol production is often reported as the overall ethanol yield (OEY, i.e. the yield calculated on the sugar content of the hydrolysate prior to detoxification and given in percent of the maximum theoretical yield) (Table 2). However, OEY does not take the relative fermentation improvement and the fermentation rate into account. A high OEY can be achieved after an intolerably long fermentation time. Since it is difficult to evaluate the significance of the improvement in fermentability without having a reference fermentation to relate it to, it is highly recommended that reference fermentations without inhibitors should be included in detoxification studies. One possibility is to evaluate the treatment on basis of the balanced ethanol yield (BEY) (Table 2) [76]. BEY is the amount of ethanol produced divided by the total amount of fermentable sugars present in the hydrolysate prior to the detoxification given as percent of a reference fermentation of a sugar solution without inhibitors.

A new development in chemical detoxification is the possibility to perform the treatment *in situ* in the bioreactor by using reducing agents, such as sulfur oxyanions or sulfhydryl reagents [39]. Reducing agents eliminate the need for an extra process step for detoxification. Furthermore, treatment with reducing agents also decreases problems with inhibition of enzymatic hydrolysis [102]. The mechanism behind treatment with sulfur oxyanions such as bisulfite and dithionite was studied by Cavka et al. [61], who found that the effect was due to sulfonation of inhibitors, which rendered them unreactive and highly hydrophilic. The substances that are sulfonated by sulfur oxyanions include phenolics [61], which is noteworthy considering indications that phenolics play a role in the inhibition of enzymatic saccharification of cellulose [21,46].

## Conclusions

Acid-catalyzed thermochemical pretreatment of lignocellulosic feedstocks has several advantages: it is a simple and inexpensive approach for pretreatment that efficiently improves the susceptibility to cellulolytic enzymes, even for more recalcitrant types of lignocellulose. A drawback is the formation of by-products that inhibit enzymes and microorganisms in subsequent biocatalytic conversion steps. However, rapid progress in several areas, such as conditioning or detoxification of slurries and hydrolysates, fermentation technology, and microbial resistance to inhibitors, makes acid pretreatment into a highly competitive future alternative in the bioconversion of lignocellulosic feedstocks. Management of inhibition problems is likely to become more important in a development that favors flexibility with respect to feedstocks, processes based on high dry-matter content and high product concentrations, and recirculation of process water.

## Competing interests

LJJ and BA are co-authors on patent applications on detoxification.

## Authors’ contributions

LJJ conceptualized, researched and wrote most of the manuscript. BA researched and wrote parts of the manuscript. NON conceptualized and critically revised the manuscript, and made most of the figures. All authors read and approved the final manuscript.
